# Identification and phylogenetic analysis of multidrug resistance, caries-related  *Streptcoccus mutans* in Sulaymaniyah City, Iraqi Kurdistan Region

**DOI:** 10.1007/s10123-026-00810-7

**Published:** 2026-04-06

**Authors:** Suha Ali Hussein

**Affiliations:** Department of Basic Sciences, College of Dentistry, Sulaimani University, Iraqi Kurdistan Region Sulaymaniyah, Iraq

**Keywords:** Phylogenetic Analysis, Multidrug resistance, *Streptococcus mutans*, Dental caries

## Abstract

*Streptococcus mutans* plays a key role in the oral ecosystem through dental plaque formation and initiation of dental caries, the foremost oral health problem around the world. This study aimed to determine antimicrobial resistance patterns and to investigate the phylogenetic relationships of local multidrug-resistant (MDR) *S. mutans* isolated from dental caries in Kurdish patients in Sulaymaniyah city, Iraqi Kurdistan Region. Dental plaque samples were collected from 160 adult Kurdish patients diagnosed with dental caries who attended the dental clinic of the College of Dentistry, University of Sulaimani. Isolation and identification of *S. mutans* were carried out using conventional culture methods, Gram staining, and the automated biochemical testing, followed by molecular confirmation using conventional PCR. Antimicrobial susceptibility testing of the confirmed isolates was performed using VITEK^®^ 2 system against seventeen antimicrobial agents. MDR isolates (resistant to ≥ 3 antimicrobial categories) were further subjected to partial sequencing of *gtfD* gene, and phylogenetic analysis (Neighbor-Joining method) was conducted by comparison with global reference strains. Out of the 160 samples analysed, 112 *S. mutans* isolates were identified, of which 13 (11.6%) were classified as MDR. Partial *gtfD* gene sequences of the MDR isolates were successfully obtained and submitted to the GenBank database. Phylogenetic analysis revealed the presence of multiple well-supported clusters, including a distinct in dependent clade as well as closely related clades showing genetic similarity to international reference strains. These findings suggest that the studied isolates represent both globally disseminated *S. mutans* lineages and region-specific genetic variants. This study demonstrates a considerably high prevalence of *S. mutans* among adult Kurdish patients with dental caries and highlights variable antimicrobial resistance patterns. The observed phylogenetic relatedness between local MDR isolates and strains from diverse geographical regions indicates overall genetic stability of *S. mutans*, with evidence of localized microevolutionary diversification. The coexistence of globally related strains and locally evolving lineages suggests ongoing international dissemination coupled with regional adaptation, underscoring the importance of continuous localized molecular surveillance and region-specific antimicrobial stewardship strategies.

## Introduction

Dental caries is a biofilm-mediated disease of the tooth, resulting from a shift in the microbial ecology of the dental plaque towards an acidogenic and acid-tolerant community (Simón-Soro and Mira 2015). It is a prevalent disease, affecting 80–90% of the global population with substantial public health impacts (Petersen 2004; Pitts et al. 2017).

Although dental caries is a polymicrobial disease, *Streptococcus mutans* remains the principle etiological agent implicated in caries initiation and progression among caries patients (Han and Vaishnava 2023), as it naturally inhabits the oral cavity, particularly within the dental plaque (Tamura et al. 2009). Among the virulence determinants of *S. mutans*, the *gtfD* gene is a conserved virulent determinant encodes glucosyltransferase D, an enzyme essential for synthesizing water-soluble glucans, which contribute to biofilm development responsible for the creation of a local acidic-microenvironment essential for its virulence expression (Xiao et al. 2012; Lemos et al. 2019; Suppiger et al. 2020). The *gtfD* gene exhibits moderate sequence variation that enables strain-level discrimination. This balance of conservation and variability makes it suitable for phylogenetic and molecular epidemiological analyses (Banas 2004; Bowen and Koo 2011).

Given the importance of biofilm formation in bacterial survival and persistence, antimicrobial resistance represents an additional major challenge. Antibiotic treatment is essential for managing and preventing complications of oral infections; nevertheless, their continued effectiveness is threatened by the increasing prevalence of bacterial resistance (Salam et al. 2023), and the antibiotics resistance genes can disperse among multispecies dental biofilm community (Sukumar et al. 2016). In traditional dental practices, antibiotic prescriptions are often empirical, with a tendency to favor broad spectrum ones (Buonavoglia et al. 2021). Furthermore, their overuse and misuse will pose a serious challenge to effective therapy and may escalate into a worldwide concern (Tenover and McGowan 1996; Almeida et al. 2020). Antimicrobial agents are commonly used for the treatment of diseases in animals, and to enhance the feed conversion ratio in livestock industries (Ezenduka et al. 2019); however, exposing the cell envelope of *S. mutans* to sub-MICs provided by antibiotics residues, found in animal products, induces the expression of *atlA* and *rgp* genes resulting in triggering the extracellular DNA-dependent biofilm formation which promote the gene exchange between resistant and susceptible bacteria within the biofilm (Dong et al. 2012; Nagasawa et al. 2020).

Despite the clinical and epidemiological importance of *S. mutans*, data regarding its identification, antimicrobial susceptibility patterns, and molecular characteristics in our region are currently lacking. Establishing baseline regional data is essential, as antimicrobial resistance patterns may vary geographically depending on prescribing practices and environmental exposure. While other cariogenic species, including *S. sobrinus*, may contribute to dental caries development, the present study focused on *S mutans* as the predominant and clinically most relevant species to provide foundational epidemiological and molecular insights in Kurdish patients in Sulaymaniyah city, Iraqi Kurdistan Region.

## Materials and methods

### Study population and sample collection

This cross-sectional study involved 160 adult patients (80 males and 80 females) with their ages ranged from 18 to 65 years, seeking treatment for an active dental caries at the dental clinic in the College of Dentistry, University of Sulaimani, from April 2024 to March 2025. This study was conducted according to the Declaration of Helsinki, and it was approved by the Ethical Committee of the College of Dentistry (Approval No. COD-EC-25-0100), and written informed consent was obtained from all participants prior to sample collection. Exclusion criteria included individuals under 18 years of age, those who were immunocompromised, and any patient who had used systemic or topical antibiotics within the last 3 months.

Dental plaque samples were collected from the intact enamel surface and cervical line of the teeth using sterile cotton swabs. Each swab was immediately placed into sterile tube containing 2 mL of Brain Heart Infusion (BHI) broth (Himedia, Mumbai, India) and transported to the microbiology laboratory for processing.

### Isolation and identification of *Streptococcus mutans*

The swabs were vortexed for several seconds within the BHI broth. An aliquot (25 µL) of the resulting suspension was then streaked on Tryptone Yeast Extract Cystine Bacitracin (TYCSB) agar (Himedia, Mumbai, India), a selective medium for *S. mutans* supplied with bacitracin (200 units/1 liter). The agar plates were incubated at 37 ◦C for 48 h under microaerophilic condition (10% CO_2_). Then the suspected bacterial colonies were subcultured on BHI agar (Himedia, Mumbai, India) supplemented with 5% of sheep blood and incubated for 48 h at 37 ◦C under microaerophilic condition. Following incubation, pure bacterial colonies showing Gram positive, chain forming cocci were isolated and loaded in VITEK^®^ 2 Compact automated system (bioMérieux, France) according to the manufacturer’s instructions, using Gram-positive identification cards (ID-GP). This system assesses 43 distinct biochemical reactions which determine the metabolic profile of each bacterial isolate.

### Confirmation of bacterial isolates by PCR assay

#### DNA extraction and quantification

Bacterial isolates of *S. mutans* identified as *S. mutans*, by VITEK^®^ 2 system, were subjected to molecular confirmation by conventional PCR. A single colony of each isolate was inoculated in 3 mL of BHI broth and incubated at 37 ◦C for 48 h under microaerophilic condition. The reference strain *S. mutans* ATCC^®^  25175^™^ was used as a positive control, and exposed to the same procedure. Bacterial pellets were obtained by centrifugation of 1.5 mL of the culture, and the genomic DNA was then extracted using Add Prep Bacterial Genomic DNA Extraction Kit (AddBioMediTek Co., Ltd, Korea). The concentration and purity of the extracted DNA were determined using NanoDrop spectrophotometer (Thermo Fisher Scientific, USA), with absorbance ratios at 260/280 nm and 260/230 nm used to assess the purity.

#### PCR amplification of *gtfD *gene

The glucosyltransferase D (*gtfD*) gene was amplified using the species–specific primers: MKD-F: (5′-GGCACCACAACATTGGGAAGCTCAGTT-3′) and MKD-R: (5′- GGAATGGCCGCTAAGTCAACAGGAT − 3′) (Hoshino et al. 2004), and the expected amplicon size is 433 bp. Each 20 µL PCR reaction contained 10 µL of 2X Add-taq master mix (AddBio, Korea), 10 pmol of each primer, and 3 µL DNA. The reaction volume was completed with nuclease-free water. The PCR was carried out in a C1000™ Thermal Cycler (Bio-Rad Laboratories, USA) with an initial denaturation step at 95 ˚C for 5 min, followed by 40 cycles of denaturation at 95 ˚C for 10 s, annealing at 60 ˚C for 30 s, extension at 72 ˚C for 20 s, and final elongation at 72˚C for 7 min.

### Visualization of PCR amplicons

The PCR products (10 µL each) were resolved by electrophoresis on 1% agarose gel in 1X of Tris acetate EDTA buffer (Inno-train, Germany). The gel was stained with ethidium bromide (Biotium, USA), and the electrophoresis was conducted at 90 V for 50 min. The resulting DNA bands were visualized using a gel documentation system (Biobas, China).

### Antimicrobial resistance analysis

Following identification, the antimicrobial resistance patterns of all *S. mutans* isolates were determined using the automated VITEK^®^ 2 Compact system and the Antimicrobial Susceptibility Test (AST-ST03) card. Bacterial suspensions were loaded into the AST-ST03 card, which automatically determines the minimum inhibitory concentration (measured by µg/mL) for 17 antimicrobial agents (Ampicillin, Penicillin G, Cefotaxime, Ceftriaxone, Chloramphenicol, Erythromycin, Clindamycin, Gentamicin, Levofloxacin, Linezolid, Moxifloxacin, Rifampin, Teicoplanin, Tetracycline, Tigecycline, Trimethoprim-Sulfamethoxazole, Vancomycin) belonging to 8 antimicrobial categories (β-lactams, macrolides, tetracyclines, amphenicol, rifamycins, fluoroquinolones, aminoglycosides and glycopeptides). The Antimicrobial Susceptibility Test card also includes a test for inducible clindamycin resistance.

### Phylogenetic analysis of the multidrug resistance *S. mutans* Isolates

Partial sequencing of the *gtfD* gene was performed for *Streptococcus mutans* isolates that showed MDR phenotype, i.e., exhibiting resistance to at least one antimicrobial agent in three or more different antimicrobial categories (Magiorakos et al. 2012). The *gtfD* gene amplicons of these MDR isolates were purified and sent to bidirectional Sanger sequencing (Macrogen, Inc., South Korea), and the resulted sequences were assembled and submitted to NCBI GenBank database to obtain the accession numbers.

The partial *gtfD* gene sequences (length ~ 432 bp) were queried against GenBank non-redundant database using the web-based Basic Local Alignment Search Tool (BLAST), and the reference sequences of the glucosyltranferase-S (*gtfD*) gene of *S. mutans* were retrieved from the GenBank database at the National Center for Biotechnology In formation (NCBI). A total of eight sequences from different countries were selected based on the complete coding sequence of *gtfD* gene and the Neighbor-Joining tree was constructed using p-distance model in MEGA software version 12 (Kumar et al. 2024). Bootstrap analysis with 1,000 replicates was performed to assess the robustness of the inferred tree topology.

### Statistical analysis

Statistical analysis to determine differences between test groups was performed using Pearson’s Chi square test and Fisher’s exact test (*p* < 0.05) in IBM SPSS Statistics for Windows, Version 31.0.

## Results

### Identification of *S. mutans*

**Fig. 1:**
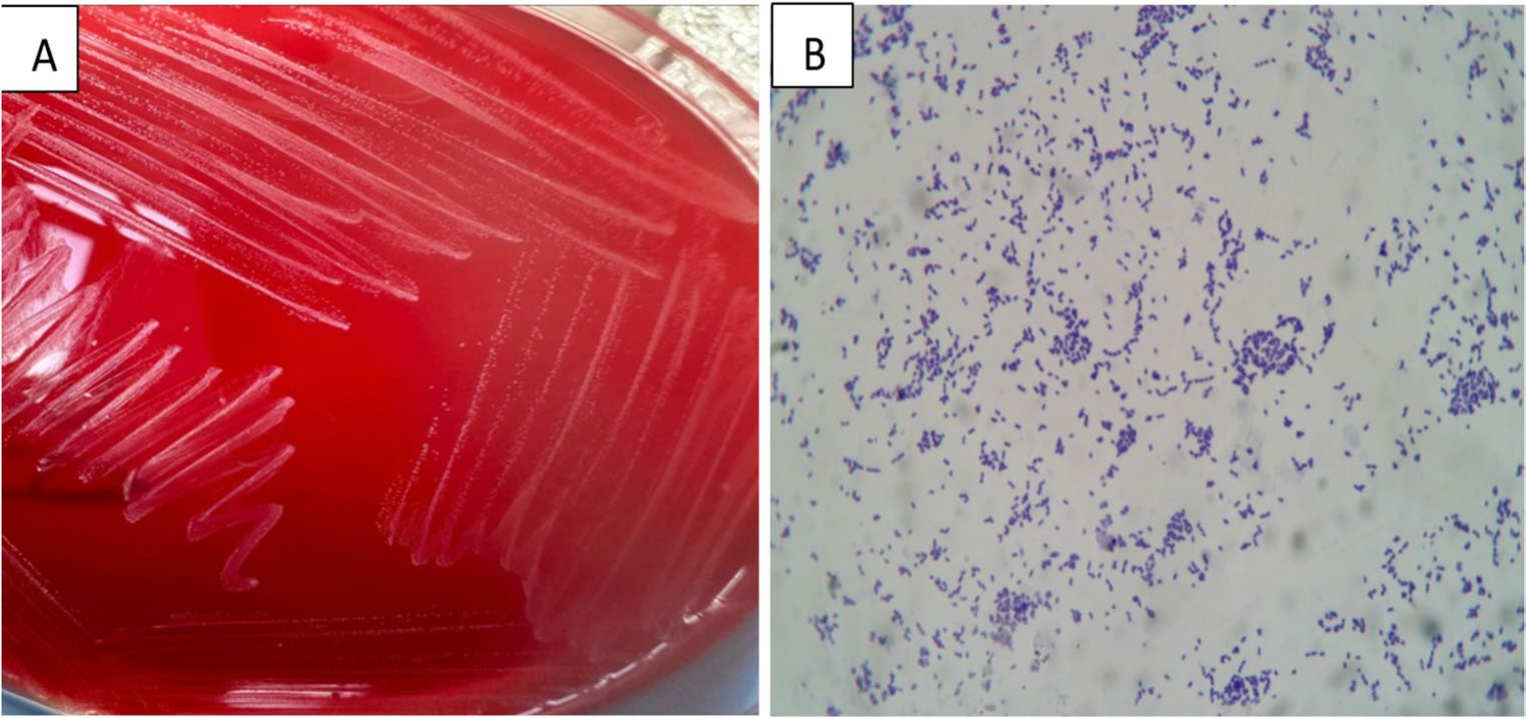
**A**. Pure colonies of *Streptococcus mutans* on BHI agar supplemented with 5% of sheep blood **B**. Gram-positive cocci of *Streptococcus mutans* forming pairs or short chains

**Fig. 2:**
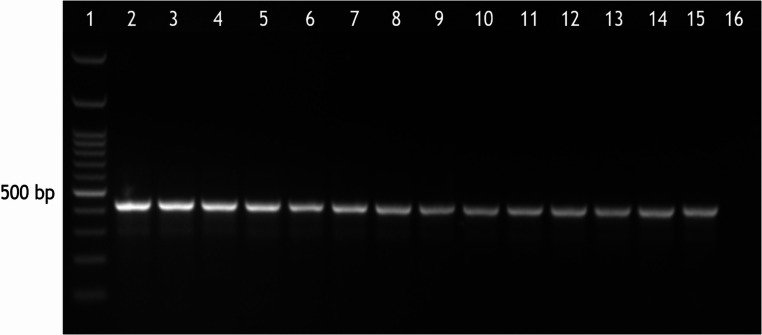
Agarose gel electrophoresis showing PCR amplification of the *gtfD* gene. Lane 1: DNA ladder, lane 2: positive control (*gtfD* gene of * Streptococcus mutans* ATCC^®^ 25175™), Lanes 3–15: *S. mutans* isolates, Lane 16: negative control (sterile double distilled water)

*Streptococcus mutans* isolates were recovered from dental plaques of 112 (70%) out of total 160 patients included in the present study. They were recovered from 54 (67.5%) of the 80 male patients and 58 (72.5%) of the female patients with no statistically significant difference (*P*- value < 0.05) between them. The isolates were identified depending on the cultural and microscopic characteristics (Fig. 1), and results of the VITEK^®^ 2 system. The molecular identification of all (112) *S. mutans* isolates were confirmed by amplification of the *gtfD* gene which resulted in the formation of 433 bp sized PCR product (Fig. 2).

### Antimicrobial resistance patterns analysis

**Fig. 3:**
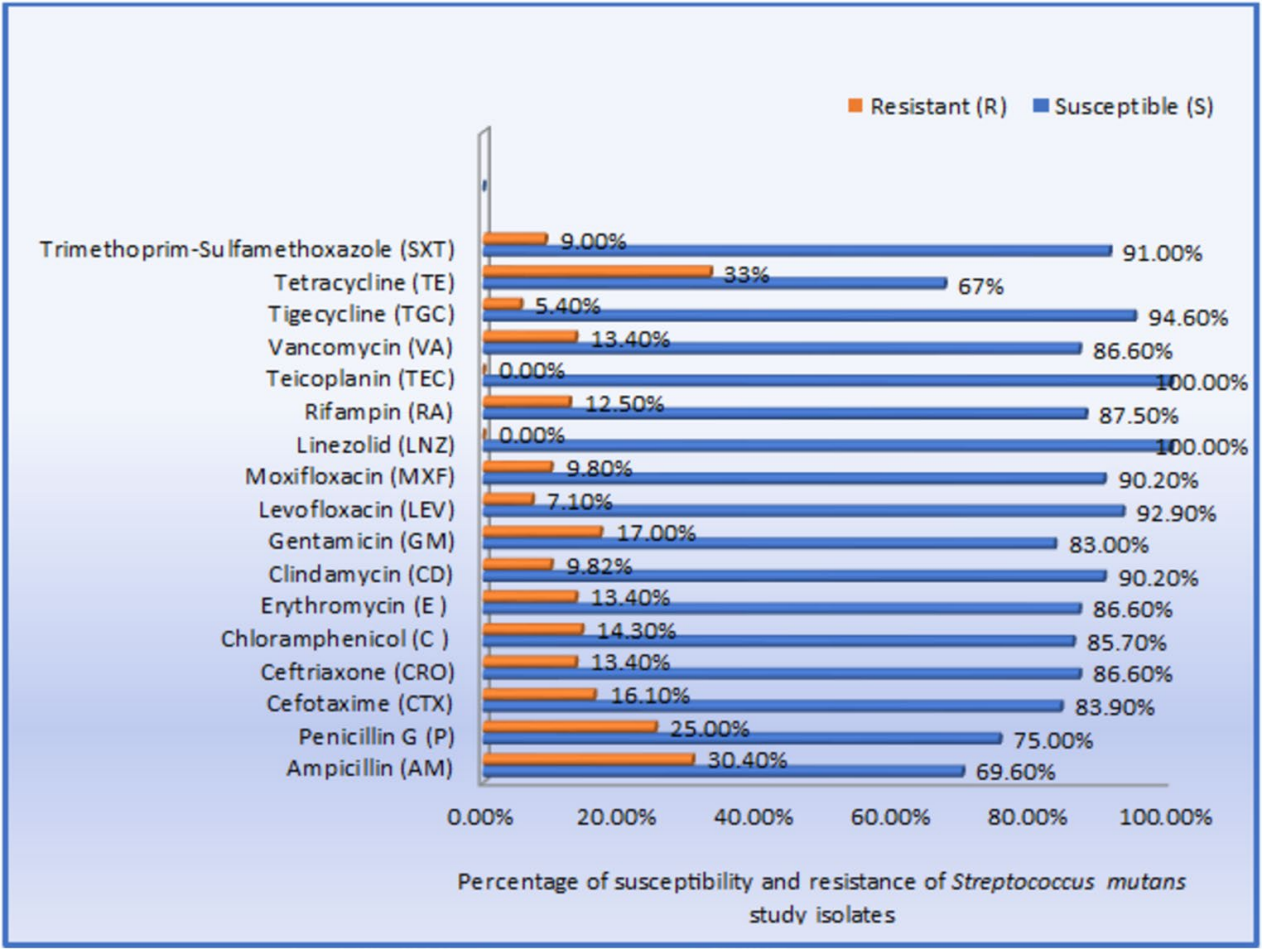
Antimicrobial susceptibility patterns of 112 *Streptococcus mutans* isolates recovered in the present study

The antimicrobial patterns of 112 *S. mutans* isolates were analyzed using a battery of 17 antimicrobial agents, which are specifically used in Vitek^2^ system for antimicrobial testing of different oral streptococci species. Variable levels of antimicrobial resistance were determined as follows in a descending order: 33% Tetracycline resistance, 30.4% Ampicillin resistance, 25% Penicillin G resistance, 17% Gentamicin resistance, 16.1% Cefotaxime resistance, 14.3% Chloramphenicol resistance, 13.4% Erythromycin resistance, 13.4% Vancomycin resistance, 13.4% Ceftriaxone resistance, 12.5% Rifampin resistance, 9.82% Clindamycin resistance, 9.80% Moxifloxacin resistance, 9% Trimethoprim-Sulfamethoxazole resistance, 7.1% Levofloxacin resistance, 5.4% Tigecycline resistance, 0.0% Teicoplanin resistance and 0.0% Linezolid resistance. All study isolates were negative to inducible clindamycin resistance (Fig. 3).

Out of the total 112 *S. mutans* isolates recovered in this study, 13 (11.61%) isolates exhibited resistance to at least one antimicrobial agent in three or more antimicrobial categories, thus meeting the definition of multidrug resistance. These 13 MDR *S. mutans* isolates were recovered from the dental plaque of 13 patients aged 50–65 years, 8 females and 5 males with no statistically significant difference (*P*- value < 0.05) between them (Tables 1 and 2).

**Table 1:** Prevalence of * Streptococcus mutans* and multidrug resistant (MDR) isolates among male and female participants

Number of Participants		Total No. of S. *mutans* isolates	No. of MDR S. *mutans* isolates
Males	80	54 (67.5%)	5 (9.26%)
Females	80	58 (72.5%)	8 (13.79%)
Total	80	112 (70%)	13 (11.61%)

MDR: Multidrugresistance referring to bacterial isolatesexhibited resistance to at least one antimicrobial agent in three or moreantimicrobial categories

No significant statistical variation was seen between numbers of *Streptococcus mutans *isolates recovered from male and female participants (*P*- value < 0.05)

### Sequencing and phylogenetic analysis of MDR *S. mutans* isolates

The *gtfD* gene amplicons of the 13 MDR *S. mutans* isolates were purified and sent to bidirectional Sanger sequencing (Macrogen, Inc., South Korea). The obtained partial sequences of the *gtfD* genes have been recorded in GenBank database under the accession numbers: PV087218 (SM-Suha-1), PV087206 (SM-Suha-2), PV087207 (SM-Suha-3), PV087208 (SM-Suha-4), PV087209 (SM-Suha-5), PV087210 (SM-Suha-6), PV087211 (SM-Suha-7), PV087212 (SM-Suha-8), PV087213 (SM-Suha-9), PV087214 (SM-Suha-10), PV087215 (SM-Suha-11), PV087216 (SM-Suha-12), and PV087217 (SM-Suha-13).

**Table 2:**
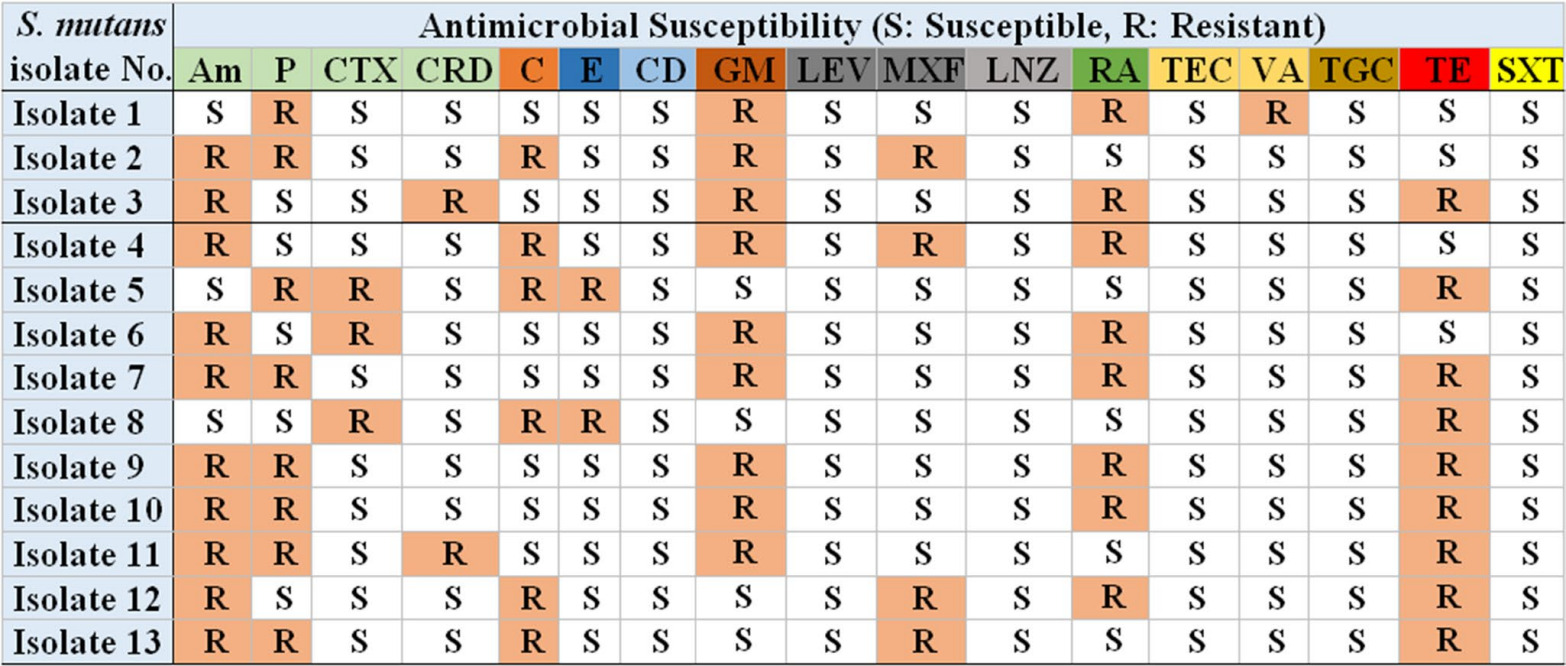
Antimicrobial resistance patterns of the MDR * Streptococcus mutans* isolates recovered in the present study

MDR: Multidrug resistance referring to bacterial isolates exhibited resistance to at least one antimicrobial agent in three or more antimicrobial categories

*AM* Ampicillin, *P* Penicillin G, *CTX* Cefotaxime, *CRO* Ceftriaxone, *C* Chloramphenicol, *E*Erythromycin, *CD* Clindamycin, *GM* Gentamicin, *LEV* Levofloxacin, *MXF*Moxifloxacin, *LNZ* Linezolid, *RA* Rifampin, *TEC* Teicoplanin, *VA*Vancomycin, *TGC* Tigecycline, *TE* Tetracycline, *SXT* Trimethoprim-Sulfamethoxazole

The obtained *gtfD* gene sequences were queried against non-redundant database in the GenBank using the web-based Basic Local Alignment Search Tool (BLAST), and the multiple sequence alignment resulted in a dendrogram in which the pairwise sequence similarity ranged from 89% to 100%. This tree revealed several well-supported clusters together with global reference strains retrieved from GenBank. The partial *gtfD* gene sequence of SM-Suha-2 and SM-Suha-13 strains demonstrated 100% sequence identity with sequences of the global reference strains JX073007.1, JX073008.1, LR134320.1 and CP101984.1; whereas the partial *gtfD* gene sequences of SM-Suha-4, SM-Suha-5, SM-Suha-6, SM-Suha-8, and SM-Suha-12 strains displayed slightly reduced genetic similarity (89–94%) to each other, and with sequences of the global reference strains JX073009.1, CP043405.1, CP013237.1, and CP044495.1.

**Fig. 4:**
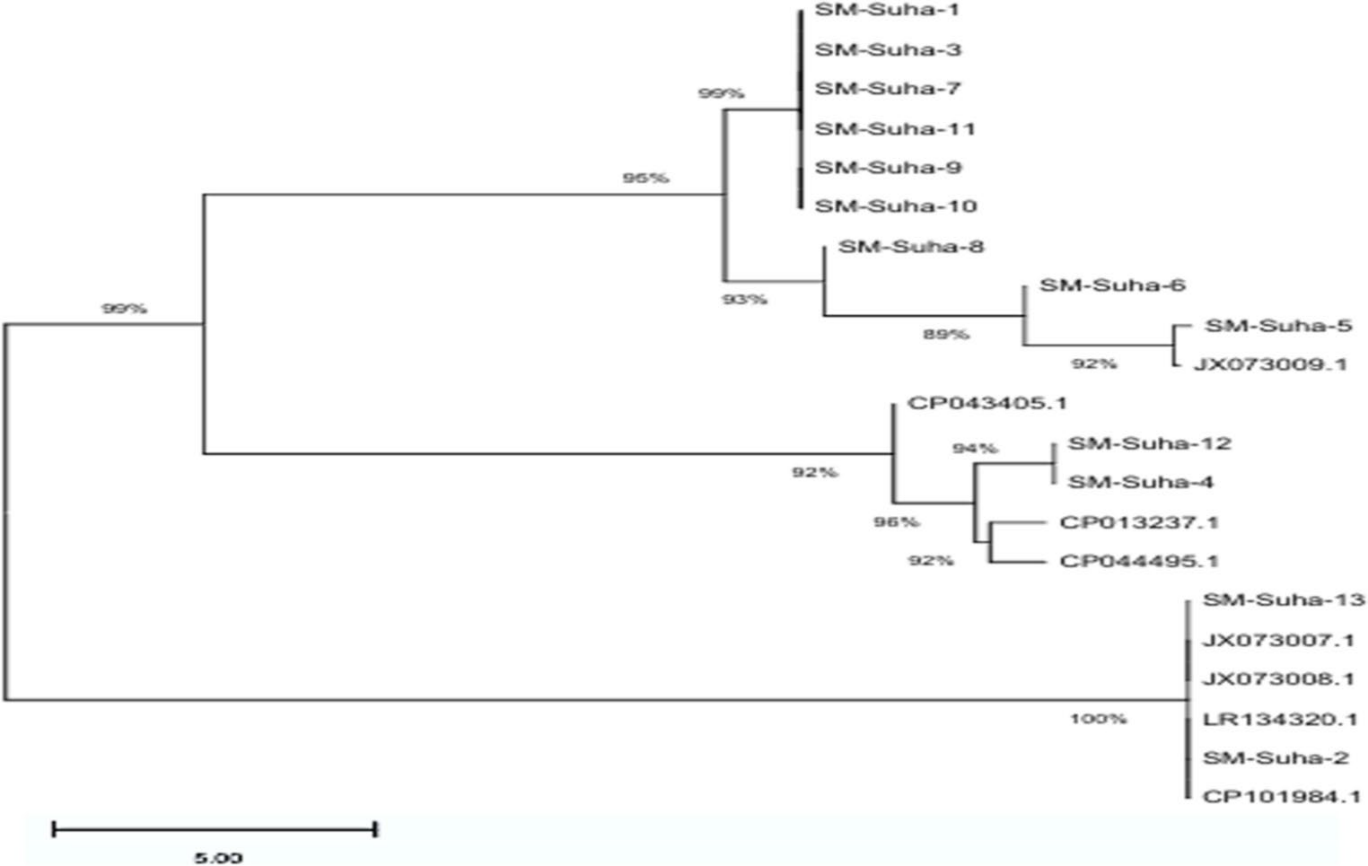
Phylogenetic tree representing the genetic relatedness between MDR strains of * Streptococcus mutans* strains recovered in this study, and global reference strains retrieved from the GenBank

On the other hand, the partial *gtfD* gene sequences of six MDR *S. mutans* strains including SM-Suha-1, SM-Suha-3, SM-Suha-7, SM-Suha-9, SM-Suha-10 and SM-Suha-11 revealed a slight but distinct sequences variation. They were almost identical (99%) to each other, but they form a separate clade not closely allied to partial *gtfD* gene sequences of any other *S. mutans* strains recovered in this study (Fig. 4).

## Discussion

Dental caries is a major public health concern affecting all age groups worldwide and *S. mutans* is still the most commonly isolated bacterial species from caries cases (Lima et al. 2020; Qudeimat et al. 2021). A considerably high prevalence of *S. mutans* isolates were recovered from patients with dental caries involved in this study (112 out of 160). This finding which is in consistent with that reported in previous studies (Okada et al. 2002; Abobakr et al. 2022), can be attributed to the positive correlation between the numbers of *S. mutans* colonizing the dental plaques and the development of dental caries (Loesche 1986).

Although statistically non-significant, females showed an increase in the prevalence of *S. mutans* (72.5%) compared to males (67.5%). This sex bias which may indicates that females are more susceptible to dental caries than males, is in agreement with findings of other authors (Ferraro and Vieira 2010; Pannu et al. 2013), and it can be ascribed to the lower salivary flow rate in females (Eliasson et al. 2006), physiological and hormonal fluctuations during menstruation and pregnancy as well as other genetic, dietary and behavioral factors (Lukacs 2011; Martinez-Mier and Zandona 2013).

The antimicrobial resistance analysis of the 112 *S. mutans* isolates recovered in this study showed a 33% Tetracycline resistance. This finding is in consistent with that recoded by Abobakr et al. (2022), and it aligns with the common opinion that this antibiotic is not recommended for the treatment of periodontal diseases because of the widespread resistance among both Gram-positive and Gram-negative bacteria (Freitas et al. 2019).

The isolates revealed variable resistance to β-lactam antibiotics, including (30.4% ampicillin, 25% penicillin G, 16.1% cefotaxime, and 13.4% ceftriaxone), consistent with previous studies (Jassam et al. 2022; Lamooki et al. 2023). Given that β-lactams remain first-line agents streptococcal infections (Bhardwaj and Kadam 2024), these findings highlight a concerning emergence of resistance. This trend is likely driven by inappropriate prescribing practices, including the combined use of penicillin and metronidazole and the overuse of β-lactams for upper respiratory infections, which exert selective pressure favoring resistant viridans group streptococci that can serve as reservoirs of resistant genes within the oral microbiota, including *S. mutans* (Nakajima et al. 2013; Goldsmith et al. 2015).

The 14.3% chloramphenicol resistance, which aligns with that reported by Karikalan and Mohankumar (2016) and Abobakr et al. (2022), is mediated by chloramphenicol acetyl transferases encoded by *cat* genes which are either chromosomally integrated or plasmid-mediated shared by streptococci (Trieu-Cuot et al. 1993). All *S. mutans* isolates recovered in this study were negative for clindamycin-inducible resistance. This finding indicates the lack of *erm* genes, which are linked to erythromycin and clindamycin resistance, in these isolates. So, the 13.4% erythromycin and the 9.82% clindamycin resistance detected in this study can be attributed to active efflux pumps and lincosamide nucleotidyltransferase activity encoded by the mobile genetic elements together with *mef* genes and *lnu* genes (Gay and Stephens 2001; Mlynarczyk et al. 2010; Chancey et al. 2015).

The MDR *S. mutans* detected in this study were mainly recovered from old patients aged 50 to 65 years. This finding may reflect age- associated alterations in the oral microbiome since the oral cavity is recognized as a reservoir of antimicrobial resistance genes that support the persistence of resistant organisms (Sukumar et al. 2023), whose composition can vary with microbial community structure and host factors (Anderson et al. 2023), as well as cumulative lifetime antibiotic exposure, repeated healthcare contact, and increased use of broad-spectrum antimicrobials associated with age-related immunosenescence (Theodorakis et al. 2024; Sukumar et al. 2024).

The partial sequences of the *gtfD* gene of the 13 MDR *S. mutans* isolates encountered in this study have been recorded in GenBank database under 13 accession numbers from PV087206 to PV087218. The phylogenetic analysis of these sequences revealed clustering within *S. mutans* tree clades with 89% to 100% similarity to each other and to sequences of global reference strains retrieved from GenBank, suggesting their clonal relationship with these strains. This finding, which reflects a high degree of conservation of the *gtfD* gene, is in agreement with Argimon et al. (Argimon et al. 2013) who denotes that the *gtfD* gene is highly conserved across geographically distinct *S. mutans* strains.

The partial *gtfD* gene sequences of the MDR *S. mutans* strains SM-Suha-1, SM-Suha-3, SM-Suha-7, SM-Suha-11, SM-Suha-9, and SM-Suha-10 were 99% identical to each other; however, they were slightly different and distinct from the other MDR *S. mutans* strains recovered in this study. The clustering of these six strains within a distinct separate clade suggests that they may represent recent descendants of a common ancestor circulating within the study area, potentially indicating local transmission within the studied population. Such phylogenetic clustering may also reflect ongoing microevolutionary diversification driven by selective pressures within the oral environment, including antimicrobial pressure and host- related factors. These evolutionary are consistent with open pan- genome structure of *S. mutans*, which promotes continuous genomic diversification and contributes to both phenotypic and virulence variations among *S. mutans* strains. This genetic flexibility not only enhances the ecological fitness of *S. mutans* but also facilitate the acquisition dissemination of antimicrobial resistance genes, enabling species to withstand antibiotic pressure and other selective challenges in the oral environment (Meng et al. 2017; Momeni et al. 2020). Future investigations incorporating resistance gene profiling and whole-genome sequencing would provide a more comprehensive understanding of the genetic determinants of antimicrobial resistance and the evolutionary dynamics of *S. mutans* strains.

## Conclusion

This cross-sectional study provides for the first time new critical data related to the prevalence of *S. mutans* and the antimicrobial resistance of its local strains among adult patients with active dental caries in Sulaymaniyah city, Iraqi Kurdistan Region. The prevalence of *S. mutans* was considerably high among dental caries patients with variable antimicrobial resistance levels against antimicrobial agents commonly used for the treatment of streptococcal infections. The identification of multiple phylogenetic clusters of MDR strains of *S. mutans* encountered in this study suggests the co-circulating of resistant lineages within our region. These findings highlight the need to promote rational antimicrobial use in dental practice through effective antimicrobial stewardship strategies to limit the emergence and spread of resistant strains, and underscore the importance of continuous monitoring of antimicrobial susceptibility patterns among oral microbiota. Further genomic studies incorporating larger sample size and advanced genomic approaches are recommended to better understand the transmission dynamics and evolutionary relationships, and public health implications of MDR *S. mutans*in the oral microbiota.

## Data Availability

Data involved in the current study can be obtained from the corresponding author upon reasonable request.
